# Exploring adults as support persons for improved pre-exposure prophylaxis for HIV use among select adolescents and young adults in the Deep South

**DOI:** 10.1371/journal.pone.0248858

**Published:** 2021-03-19

**Authors:** Samantha V. Hill, Jarvis Johnson, Fazlur Rahman, Emily F. Dauria, Michael Mugavero, Lynn T. Matthews, Tina Simpson, Latesha Elopre

**Affiliations:** 1 Department of Pediatrics, The University of Alabama at Birmingham School of Medicine, Birmingham, Alabama, United States of America; 2 Division of Infectious Diseases, Department of Medicine, The University of Alabama at Birmingham, Birmingham, Alabama, United States of America; 3 Department of Biostatistics, The University of Alabama at Birmingham, Birmingham, Alabama, United States of America; 4 Division of Infant, Child and Adolescent Psychiatry, The University of California San Francisco, San Francisco, California, United States of America; 5 Department of Medicine, The University of Alabama at Birmingham, Birmingham, Alabama, United States of America; Ohio State University, UNITED STATES

## Abstract

**Purpose:**

Pre-exposure prophylaxis for HIV (PrEP) is an effective yet underutilized biomedical tool for adolescents and young adults’ (AYA) HIV prevention due to barriers such as PrEP adherence. We assessed HIV prevention knowledge, attitudes and beliefs from adults who self-identified as a primary support person to an AYA.

**Methods:**

We surveyed AYA primary support persons at an academic hospital. Univariate and multivariate regression analyses were completed to identify factors associated with the belief AYAs engaging in HIV-associated behaviors should use PrEP and willingness to support AYAs on PrEP.

**Results:**

200 primary support persons completed the survey. Participants were predominately female (77%) and black (56%). Nearly all primary support persons believed AYAs engaging in HIV-associated behaviors should take PrEP (94%) and 98% would support an AYA taking PrEP via transportation to appointments, assistance with refilling prescriptions, medication reminders, or encouragement.

**Conclusions:**

Primary support persons are willing to support AYAs using PrEP.

## Introduction

The Southern United States (U.S.) accounted for 52% (19,968) of new HIV diagnoses in 2018 [1]. Nearly a quarter (21%) were among adolescents and young adults (AYA) ages 13 to 24 [1]. In Alabama, AYAs account for 31% of new cases [2]. Numerous HIV prevention tools, such as consistent condom use and pre-exposure prophylaxis for HIV (PrEP), could address these disproportionate rates but remain underutilized by AYAs [3]. Despite the expanded indication in 2018 for PrEP (tenofovir emtricitabine or TDF/FTC) for anyone weighing at least 35kg (~77lbs), there remain numerous barriers to increasing AYA PrEP use including low PrEP knowledge, challenges adhering to a daily medication, medication costs, HIV- and sex-related stigma, parent and provider concerns, and confidentiality [3–6]. For instance, demonstration studies among AYAs highlight adherence challenges as only 22% of 15 to 17 year olds were adherent to PrEP over a 48-week period, with adherence declining after visit spacing was increased from monthly to quarterly [7]. Similar rates of poor adherence have also been seen among 18 to 22 year olds in the U.S. [8]. Strategies that promote effective use of PrEP among AYAs with behaviors associated with high HIV incidence are desperately needed.

**Fig 1:**
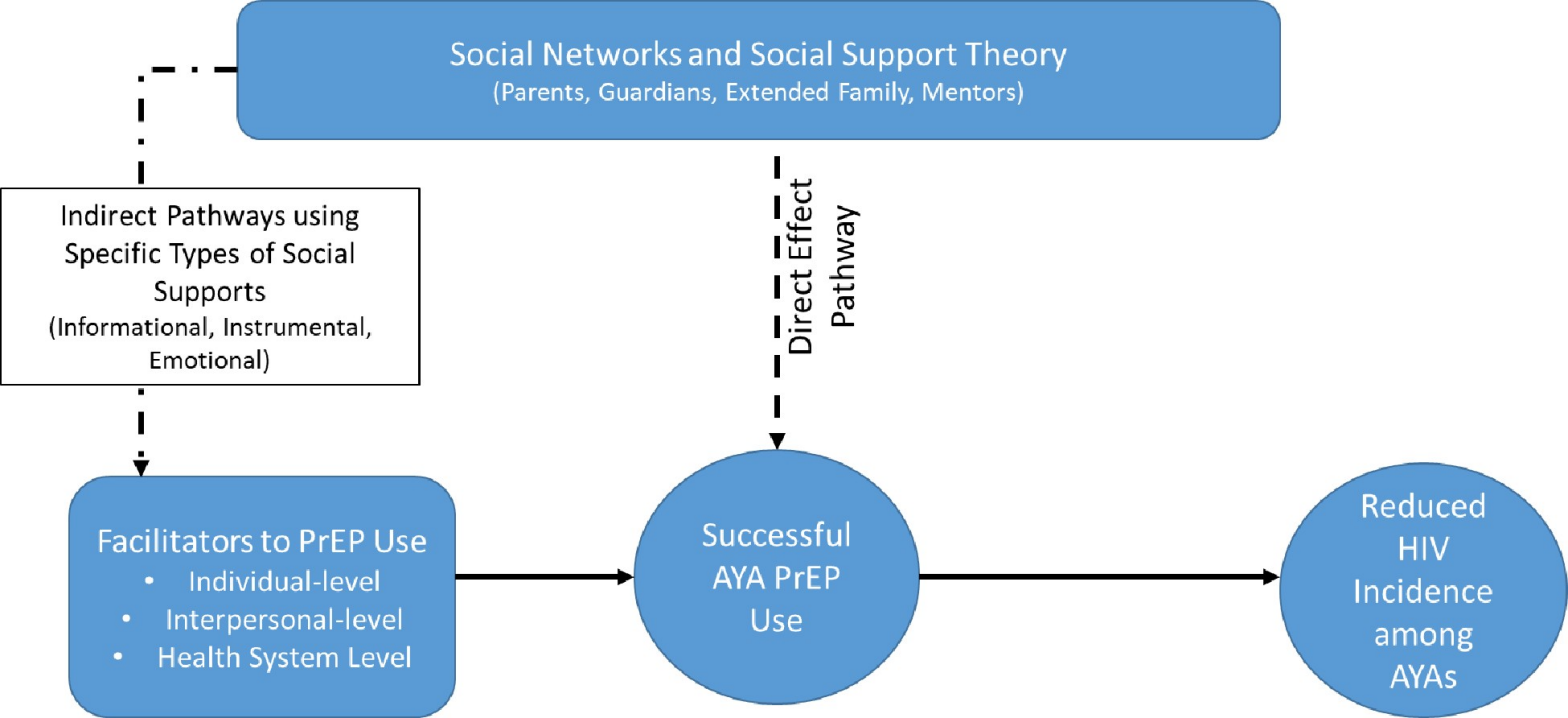
Social networks and social support theory direct and indirect pathways. Fig 1 illustrates how social support may directly (Direct Effect Pathway) improve AYA PrEP use or indirectly aid in removing barriers to PrEP use (Indirect Pathways) among AYAs by providing informational, instrumental, and emotional types of social support.

One such strategy involves engaging parents and other adult caregivers to support AYAs with PrEP. The Social Networks and Social Support (SNSS) Theory (Fig 1) postulates that provision of informational (e.g., medication reminders), instrumental (e.g., assistance with paying for medications or transportation to visits), and emotional (e.g., encouragement) support can improve relationships between people and indirectly affect behavior [9]. Research has shown that informational support provided by parents assists AYAs with adherence to daily medication regimens for chronic conditions such as HIV and diabetes mellitus [10–14]. Additionally, parents may help to promote safer sex and healthy relationships among AYAs by providing informational support [15,16]. While many AYAs may not feel comfortable discussing their sexual health with parents, studies show that providing parents with the skills to engage AYAs in conversations about sex can promote discussions around safe sex and healthy partnerships [17,18]. There is also evidence that this type of support may translate to AYAs’ utilization of PrEP. A study that examined African American maternal figures’ knowledge and attitudes about PrEP use among their daughters (≤18 years) found that maternal figures’ attitudes were favorable towards PrEP [19]. Another study of AYA-parent dyads found that both parties were likely to be accepting of PrEP use among adolescents [20]. Neither study, however, examined adult attitudes’ towards assisting AYAs with PrEP adherence. To our knowledge, there are no studies evaluating adult caregivers’ attitudes towards providing informational, instrumental, or emotional support to AYAs using PrEP.

We sought to address this gap in the literature by exploring if AYA caregivers (e.g. parents, guardians, aunts, uncles, grandparents) were open to providing social support to assist AYAs in PrEP adherence. We conducted a cross-sectional study of AYA caregivers to assess their knowledge of HIV risk, HIV prevention, and PrEP, and ways in which AYA caregivers believe they might be able to assist AYAs with PrEP. We hypothesized that caregivers with greater knowledge about HIV, HIV prevention, and PrEP would be more willing to support an AYA with PrEP use by providing social support.

## Materials and methods

### Study population and setting

We recruited caregivers of AYAs aged 11 to 21 years from an academic university hospital located within an urban setting in the Deep South between January and June 2019. A convenience sample was recruited from waiting areas in an adolescent clinic and a hospital lobby. The adolescent clinic provides general and subspecialty services to AYAs in this age group, including confidential services pertaining to sexual health. Demographics of patient populations served within the adolescent clinic include 70% female, 85% Black, 80% on public insurance, and 70% living in urban and surburban settings. (It is not customary to collect data of parents or guardians who accompany adolescents to the clinic.) The university hospital lobby is within the adjacent adult hospital that is often frequented before or after individual appointments or by families accompanying individuals to appointments. (The demographics the university serves represent the demographics for the state and are characterized as 52% female, 68% White, 17% on public insurance, and 56% living in rural areas [21–23].) We initially recruited from the adolescent clinic and then expanded data collection to the hospital lobby to decrease possible sampling bias associated with being a caregiver willing to bring an AYA to a medical appointment. Study personnel actively approached any adult accompanying an AYA within the clinic waiting room or the hospital lobby. Inclusion criteria were self-identification as a parent, primary caregiver, or primary support person of an AYA between 11–21 years old, henceforth referred to as primary support persons. Primary support persons were individuals who self-reported supplying informational (e.g. providing information on how to remain safe), emotional (e.g. providing encouragement), and/or tangible support (e.g. providing money, transportation) to AYAs and included parents, guardians, adult siblings, extended family, and adult support figures. Exclusion criteria included inability to understand written or spoken English. Informed consent was obtained verbally prior to the completion of a self-administered electronic survey (or survey questions were read verbatim for individuals with visual impairment or self-reported literacy challenges). All surveys were completed in a quiet secluded area of the clinic or lobby and participants received a $15 gift card for their participation. This study was approved by the University of Alabama at Birmingham Institutional Review Board on 11/16/2018. No approval number was obtained. Participants were provided an information sheet with descriptions of the study purpose and procedures. Documented consent was waived for this study due to the use of the informational sheet at the beginning of the survey and the collection of data anonymously. For individuals with visual impairment or literacy challenges, the information sheet was read verbatim.

### Survey design

The 36-item survey was divided into four sections (S1 Appendix). Estimated completion time was 8 minutes. Participants were asked to complete the survey with reference to the oldest AYA in their care or for those participants who were non-parent/guardian caretakers, the AYA in their care for whom they felt this study would be most applicable. The first section (Q1-15) contained items adapted from a survey assessing the knowledge, attitudes, and beliefs regarding general HIV-related knowledge and prevention among U.S. college students [24]. The Cronbach’s alpha scale was >0.90 [24]. These questions were scored as low (<24 correct), average (24–28), or high (≥29) HIV knowledge as described elsewhere [24]. The second section (Q16-Q21) contained: A) a brief vignette about PrEP including what it is and what it is used for, B) questions regarding the participant’s attitude about AYA use of PrEP using a 4-point Likert scale (choices being agree strongly, agree, disagree, and disagree strongly), and C) questions assessing willingness to support an AYA using PrEP. Participants were given four options in which they might see themselves supporting an AYA on PrEP: rides to appointments, medication reminders, money, and encouragement. Questions for section 2B were adapted from emergent themes from a qualitative study exploring parental support of daily medication administration among parents of AYAs with cystic fibrosis [25]. The third section (Q22-Q27) of the survey assessed self-efficacy with respect to assisting with AYAs’ health and PrEP care using the same 4-point Likert scale as above. These questions were also adapted from a qualitative study exploring medication adherence in AYAs with cystic fibrosis and their parents [25]. The fourth section (Q28-35) asked demographic questions (e.g., age, gender identity of primary support persons and their AYA) and a question about if primary support persons knew whether their AYA was sexually active (definitely agree, agree, disagree, definitely disagree, unsure). The survey did not ask for respondent to report the sexual identity or gender identity of the AYA. All surveys were anonymous and collected and stored using SurveyMonkey®.

### Study outcomes

The primary study outcomes were belief that an AYA at-risk for HIV should take PrEP and willingness to support an AYA on PrEP (both of which were dichotomous variables). Primary support person’s knowledge of HIV and prevention, knowledge of PrEP, self-efficacy, and demographics were included as independent variables to determine their association with the study outcomes.

### Statistical analyses

The sample size was determined based on data from a prior study illustrating approximately 44% of 429 adolescents accessing care at the same adolescent clinic within the current study having an indication for PrEP [26]. (At the time this study was designed there was not information available to conduct a formal power analysis.) We conducted descriptive analyses to characterize the study participants. For Likert responses, we grouped agree with strongly agree and disagree with strongly disagree responses, respectively. We performed Chi-square, Fisher exact, Mann Whitney U, Student’s-t or Kruskal-Wallis tests as appropriate for initial univariate primary outcomes analysis. No models were developed to analyze primary support persons’ willingness to support an AYA on PrEP because there was limited variability in responses: 98% of participants reported they would be willing. We provided descriptive data for our sub-analysis of primary support person’s willingness to support an AYA on PrEP based on AYA’s insurance, primary support person’s race and sex and primary support person’s confidence in their ability to help an AYA make recommended lifestyle changes. We used logistic regression models to assess the covariates associated with dichotomous outcomes. To quantify the effects of covariates of interest, we estimated odds ratios with 95% confidence intervals. Prior to final multivariate analyses, we performed a stepwise initial variable selection and variables with p-value less than or equal to 0.15 were initially included in the multivariate models. All hypothesis tests were two-tailed and we used a p-value < 0.05 to indicate statistical significance. We performed analyses in SAS for Windows® version 9.4 (Cary, NC).

## Results

### Survey responses

Between January and June 2019, 250 individuals were approached to complete the survey and 200 individuals completed the survey (~80% enrollment rate). Lack of guardianship or relationship with an AYA that met study criteria were two main reasons for ineligibility (data not presented). Reasons why eligible participants declined study enrollment included lack of interest or lack of time to complete survey (data not captured). We recruited 150 participants from the adolescent clinic and 50 participants from the hospital lobby. As shown in Table 1, primary support persons were predominantly female (77.3%), with 32.5% between ages 36 and 45 years old. Fifty-six percent self-reported a Black/African-American racial identity and nearly all were non-Hispanic (99.5%). Most reported being a primary support for a female AYA (61.0%) and an AYA between 16–21 years old (51.8%). Demographics from the two locations only differed with regards to greater percentages of Black/African American racial identity and referent AYAs with private insurance among participants from the hospital lobby when compared to participants recruited from the adolescent clinic.

**Table 1:** Primary support person demographics (N = 200).

Demographic		Total Participants	Hospital Lobby	Adolescent Clinic
		N = 200	N = 50	N = 150
		N (%)	N (%)	N (%)
**Primary Support Person’s Age^a^ (in years)**				
	30 or younger	30 (15.2)	8 (16.3)	22 (14.9)
	31–35	27 (13.7)	5 (10.2)	22 (14.9)
	36–45	64 (32.5)	15 (30.6)	49 (33.1)
	46–54	50 (25.4)	11 (22.4)	39 (26.3)
	55 or older	26 (13.2)	10 (20.4)	16 (10.8)
**Primary Support Person’s Gender^a^**				
	Woman	153 (77.3)	37 (74.0)	116 (78.9)
	Man	44 (22.2)	13 (26.0)	30 (20.4)
	Transgender woman	1 (0.5)	0 (0.0)	1 (0.7)
**Primary Support Person’s Ethnicity**				
	Hispanic	1 (0.5)	0 (0.0)	1 (0.7)
	Non-Hispanic	196 (99.5)	49 (100.0)	148 (99.3)
**Primary Support Person’s Race^a^**				
	American Indian/Alaskan Native	2 (1.0)	0 (0.0)	2 (1.3)
	Asian	1 (0.5)	1 (2.0)	0 (0.0)
	Black/African American	110 (55.3)	34 (69.4)	75 (50.7)
	Caucasian	86 (43.2)	14 (28.6)	71 (48.0)
**Referent AYA Age^a^**				
	11–15	93 (48.2)	25 (55.6)	101 (68.7)
	16–18	78 (40.4)	13 (28.9)	31 (21.1)
	19–21	22 (11.4)	7 (15.5)	15 (10.2)
**Referent AYA Sex^a^**				
	Female	119 (61.0)	29 (63.0)	90 (60.4)
	Male	76 (39.0)	17 (37.0)	59 (39.6)
**Referent AYA Insurance^a^**				
	Public	84 (42.6)	11 (22.4)	73 (49.3)
	Private	104 (52.8)	33 (67.3)	71 (48.0)
	None	9 (4.6)	5 (10.2)	4 (2.7)

^a^Missing demographic responses Total Participants N(%): primary support person age-3 (1.5); primary support person gender-2 (1.0); primary support person ethnicity-3 (1.5); primary support person race-1 (0.5); AYA age-7 (4.0); AYA sex-5 (3.0); AYA insurance-3 (1.5); Knowledge-7 (4.0); Hospital Lobby N(%): primary support person age-1(2.0); primary support person gender- 0(0.0); primary support person ethnicity- 1(2.0); primary support person race-1(2.0); AYA age-5(10.0); AYA sex-4(8.0); AYA insurance-1(.0); Adolescent Clinic N(%): primary support person age- 2(1.3); primary support person gender- 3(2.0); primary support person ethnicity- 1(0.7); primary support person race-2(1.3); AYA age-3(2.0); AYA sex-1(0.7); AYA insurance-2(1.3).

### Outcomes of interest

**Fig 2:**
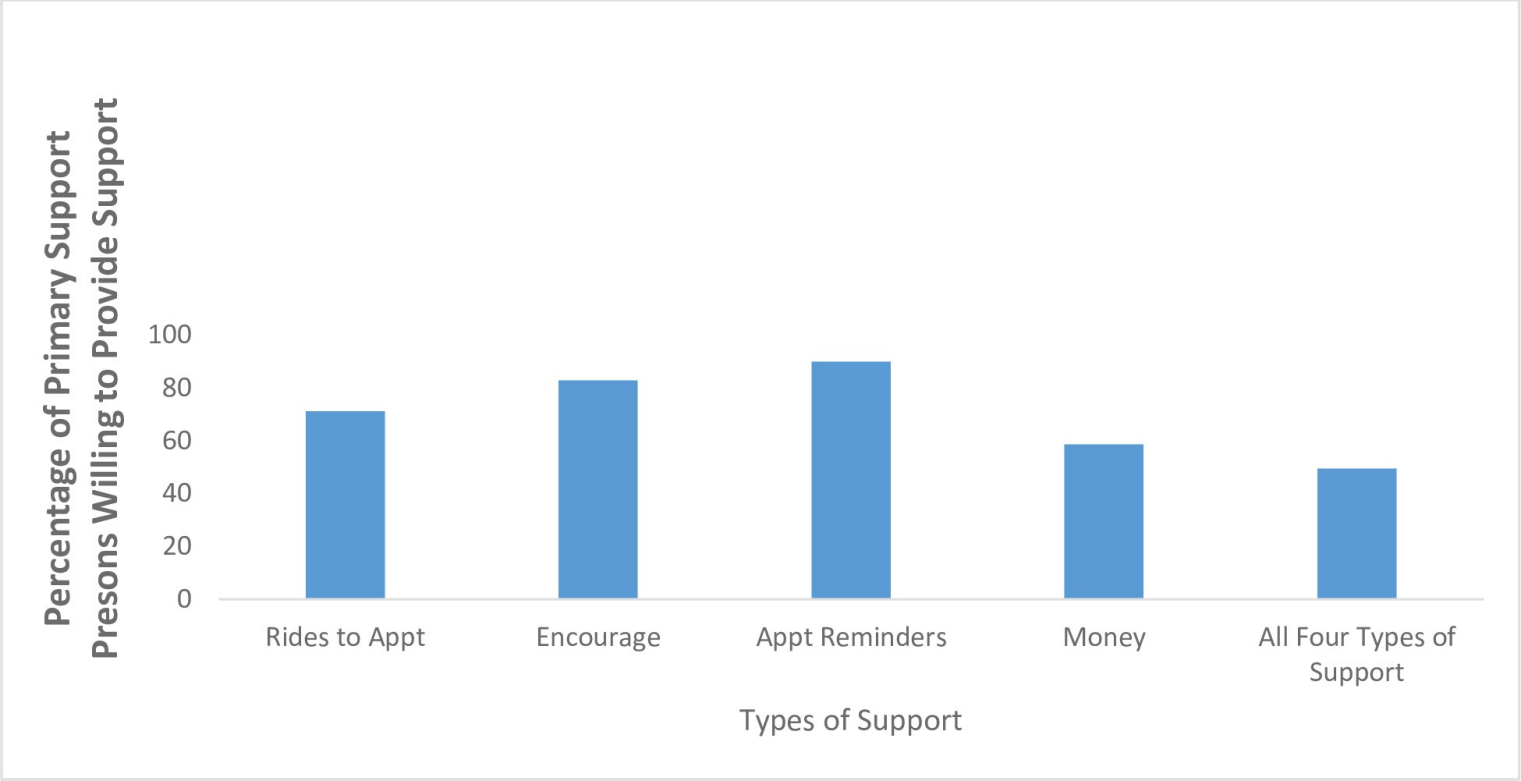
Manner in which primary support persons are willing to support AYAs on PrEP. Fig 2 illustrates ways in which primary support persons were willing to support AYAs on PrEP (including combinations of types of support). Each manner of support (e.g. transport) includes the percentage of primary support persons who were willing to provide that manner of support by itself or in combination with any of the other types of support.

**Fig 3:**
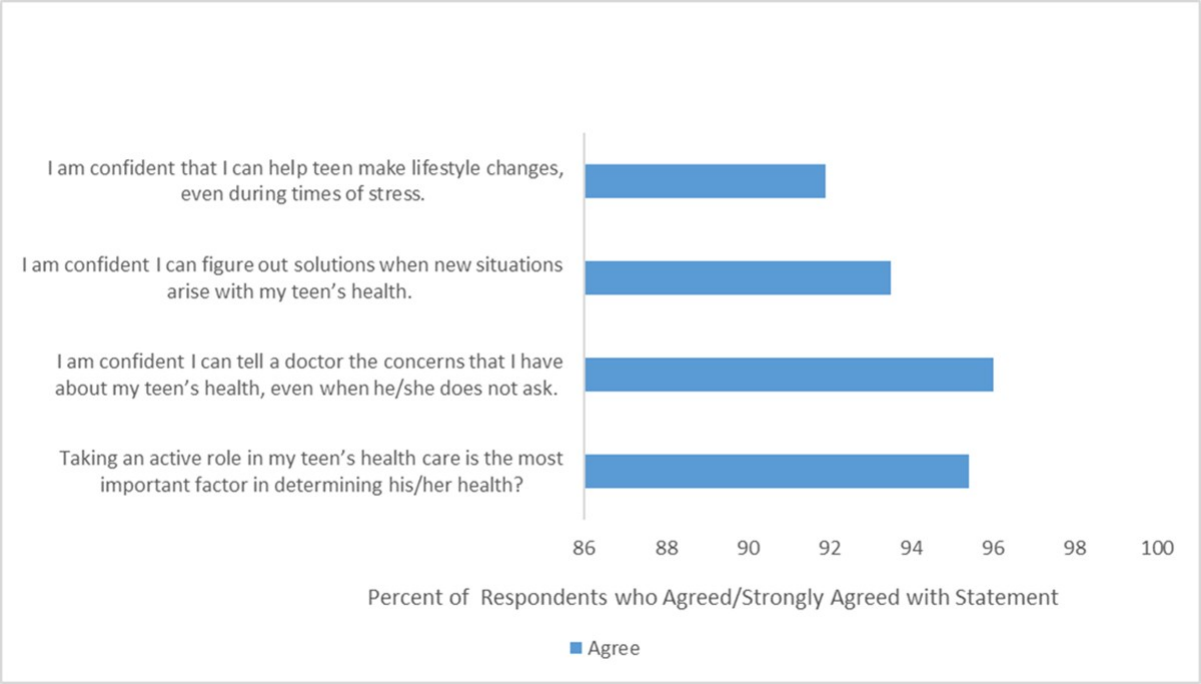
Primary support Person’s self-efficacy in affecting AYA’s health. Fig 3 illustrates primary support person’s self-efficacy in how they may impact AYAs’ health.

Overall, 186 (94%) primary support persons believed that an AYA at risk for HIV should take PrEP and 196 (98%) primary support persons reported willingness to support an AYA with PrEP if the AYA asked them for support (Table 2). Primary support persons who identified as women were more likely to believe AYAs should take PrEP in adjusted models (aOR 5.5, 95% CI 1.62, 18.89) (Table 3). In addition, 99 (49.5%) primary support persons had low HIV knowledge with 94 (47.0%) and 7 (3.5%) primary support persons having average, and high HIV knowledge, respectively. All primary support persons with high HIV knowledge believed AYAs at risk for HIV should take PrEP; however 99% and 89% of primary support persons with average and low HIV knowledge respectively believed AYAs at risk for HIV should take PrEP (p = 0.01). Responses were similar between the two locations with 94% of primary support persons from the adolescent clinic believing an AYA at risk for HIV should take PrEP compared to 96% from the hospital lobby; 98% of primary support persons from both locations report willingness to support an AYA with PrEP. Among 198 participants willing to support an AYA with PrEP, almost half reported being willing to provide all four types of support (Fig 2). The most common types of individual support included providing reminders to take medication (178, 90%) and encouragement (164, 83%) (Fig 2). African-American primary support persons were less likely to reporting willingness to provide medication reminders (OR 0.2 95% CI 0.05, 0.58) and financial support (OR 0.6 95% CI 0.32, 1.0) in unadjusted models. Fig 3 illustrates primary support persons’ attitudes towards their self-efficacy in assisting AYAs in routine parts of a healthcare visit and incorporating recommendations from that visit into their lives.

**Table 2:** Primary support person responses to select survey questions.

Select Survey Questions	Response	N (%)
**Knowledge that AYA is sexually active**		
	Agree	67 (34.7)
	Unsure	28 (14.5)
	Disagree	98 (50.8)
**General HIV Knowledge (Q1-15)**		
	High	7 (3.5)
	Average	94 (47.0)
	Low	99 (49.5)
**Primary Support Person’s Knowledge and Attitudes about PrEP**		
**There is a medication that is effective in reducing a person’s chance of getting HIV**		
	True	121 (60.8)
**Have you heard of PrEP before today?**		
	Yes	68 (34.0)
**If a teen were at high risk of becoming infected with HIV, do you think he/she should take this medication**		
	Yes	186 (93.9)
**If a teen were prescribed PrEP, do you think he/she would be responsible in taking it as prescribed without the assistance of an adult?**		
	Yes	90 (45.0)
**If a teen asked for your support with them taking PrEP would you support them?**		
	Yes	196 (98.0)

^a^Participants selected all manners they were open to supporting an AYA.

Missing responses to select questions N(%): Q16-1 (0.005); Q18-2 (0.01); Q21-2 (0.01).

**Table 3:** Select univariate and multivariable analyses for primary support Person’s knowledge, attitudes and beliefs (KAB) about PrEP.

Primary Support Person’s KAB Outcome of Interest		Predictor	OR (95% CI)	aOR (95% CI)^b^
**Belief that AYAs at-risk for HIV should take PrEP**	AYA Insurance	Private	**5.1 (1.22, 21.39)**	**4.4 (1.03, 19.24)**
		No Insurance	2.1 (0.10, 47.50)	2.6 (0.10, 67.55)
		Medicaid	Ref	Ref
	Primary Support Person’s Race	African American	0.2 (0.05, 1.09)	0.2 (0.04, 1.23)
		Non-African American	Ref	
	Primary Support Person’s Sex	Female	**3.5 (1.08, 11.55)**	**5.5 (1.62, 18.89)**
	Primary Support Person’s Sex	Male	Ref	Ref
	Confidence in ability to help AYA make Recommended lifestyle changes	Agree	2.4 (0.49, 12.26)	
		Disagree	Ref	

^a^AYA = adolescent and young adult.

^b^Adjusted models- AYA Insurance, Primary Support person’s race and sex.

## Discussion

AYAs are disproportionately affected by HIV in the South, and Alabama specifically [1,2]. Although PrEP is effective at preventing HIV including among AYAs, PrEP uptake among AYAs in the South is low [27]. Given that many AYA PrEP implementation barriers pertain to accessing care, adhering to medication and appointment regimens, and feeling supported in taking medicine, we grounded this study in the Social Networks and Social Supports (SNSS) theory postulating that direct and indirect social supports from parents, guardians, and other trusted adults (i.e. primary support persons) may be a potential solution to improving PrEP success among AYAs in the South. We found that when posed with a hypothetical situation where an AYA aged 11 to 21 approached a primary support person for support, 98% of primary support persons were willing to support an AYA on PrEP, with 60–90% of participants willing to provide a combination of monetary support, transportation, encouragement, and medication reminders. Primary support persons were willing to provide these supports and had high levels of self-efficacy to do so, even though more than half did not think their AYA was engaging in behaviors known to be associated with HIV acquisition.

Literature from chronic conditions such as cystic fibrosis and type 2 diabetes supports incorporation of primary support persons into AYA healthcare as a strategy to improve adherence to medication regimens and associated health outcomes [10–14]. These same findings have been described among AYAs living with perinatally-acquired HIV [28]. Furthermore, HIV literature suggests that non-related support persons can be beneficial supports, particularly with groups of individuals who may feel marginalized [19,29–31]. Project nGage found men who have sex with men ages 16–29 at-risk for HIV who had a supportive confidant (e.g., friend, family member) had greater rates of PrEP visit attendance and retention in care [31]. While AYA-parent dyadic communication is the preferred standard for sexual health discussions, studies continue to illustrate the need and importance of including non-parental figures into AYA sexual health discussions.

The positive impact that support persons have on emerging adults may be further magnified among younger populations. The continual development and maturation of the pre-frontal cortex until the age of 25; real and perceived stigma pertaining to HIV, PrEP, sex, and sexuality; and fewer physical resources (e.g. money, transportation) contribute to poor uptake of and retention in PrEP care for AYAs [29]. Specifically, the stigmas related to sexual health are amplified in places like Alabama and the South where cultural and social norms, including religious and political views, discourage conversations about sexual orientation, sexual behaviors, and experiences with stigma associated with an individual’s sexuality [32]. The silence around sex and sexuality is also present within the healthcare infrastructure, in some instances, creating barriers to client-provider discussions of PrEP [32]. Engaging a support person can provide a mechanism to supplement AYAs with the resources they may lack. The Direct Effect Pathway for Social Support from the SNSS theory postulates that provision of support and love to the AYA can mitigate circumstances where the AYA may feel isolated due to community stigma with HIV, sex, or being a gender or sexual minority [9,33]. Indirect Pathways of the SNSS theory illustrate how support persons can provide informational support such as knowledge regarding why adhering to PrEP and clinic visits is beneficial and important; instrumental support such as providing rides to appointments and assistance with paying for medications or clinic visits; or emotional support such as encouragement with taking medications and reduction of sex-related stigma [9,33]. Currently studies focused on deploying primary support persons to foster AYA PrEP adherence in the South are lacking.

This study should be understood in the context of several strengths and limitations, which point to important areas of future research. Strengths include the gap in the literature this study addresses related to understanding factors shaping PrEP expansion and uptake among AYAs in the Southern U.S. This study was conducted in a state with a population at high-risk of HIV transmission, an under-resourced geographic hotspot as identified in the Department of Health and Human Services’ *Ending the HIV Epidemic* plan focused on reducing HIV incidence by 90% in 10 years [34,35]. In addition, these findings provide support for the acceptability and willingness of a primary support person to assist AYAs with PrEP adherence. The study also used a broad definition of primary support persons, encompassing individuals beyond AYAs’ parents/guardians. Leveraging primary support persons to support PrEP use among AYAs has the potential to expand uptake and access of PrEP among AYAs with indications for PrEP and reduce regional disparities in national HIV incidence.

Individuals participating in the present research study represent a convenience sample, and therefore results do not represent all individuals who provide primary support to AYAs. Participants were recruited from a healthcare setting and the attitudes and perceptions of primary support persons actively engaged in healthcare may differ from those recruited from community settings. While participants had low HIV knowledge, their support for PrEP was high which may reflect a less informed endorsement of support. In addition, survey items regarding support for AYAs asked participants to reflect on PrEP use and support for the oldest AYA to whom participants were guardians or the most relevant AYA for non-parental support persons. Thus, primary support person’s perception of support may differ depending on the age of the AYA in the primary support person’s care. Additionally, select survey items inquired about hypothetical AYAs, which may have influenced responses. While a large percentage of primary support persons were agreeable to supporting a hypothetical at-risk AYA on PrEP, only a small percentage of primary support persons believed their referent AYA was sexually active. Primary support persons may have reported supporting PrEP use because of a social desirability bias. In addition, supporting PrEP use for a hypothetical teen poses a low opportunity cost for a primary support person who does not believe their AYA is sexually active. Future studies should tailor survey questions to inquire specifically about the attitudes and perceptions of AYAs in primary support persons’ care and/or could include support persons and the referent AYA as a dyad.

Additionally, our study centered on examining knowledge and attitudes of primary support persons and not AYAs themselves. Future research should explore AYAs’ perceptions of how primary support persons may promote PrEP uptake and adherence. Potential areas of future work include examining from whom AYAs would like to receive support in adhering to PrEP medication and the associated care regimen, the ways in which support would best be provided, and desired communication strategies for discussing and negotiating support in PrEP uptake and adherence.

## Conclusion

Primary support persons enrolled from health care settings in the Deep South are interested in supporting AYAs to use PrEP for HIV prevention. More research is needed on how to engage primary support persons and their associated AYAs to promote PrEP use for this population with a high unmet need for PrEP and disproportionate burden of new HIV infections.

## Supporting information

S1 Table De-identified raw data. This contains all de-identified collected data.
(XLSX)

S1 Appendix Participant survey. This is the survey that was administered to all participants included in this study.
(PDF)
