# Supplementary material for: Exploring adults as support persons for improved pre-exposure prophylaxis for HIV use among select adolescents and young adults in the Deep South
Source: PLoS One. 2021 Mar 19;16(3):e0248858. doi: 10.1371/journal.pone.0248858 (PMC7978356; doi:10.1371/journal.pone.0248858)
Supplement: S1 Appendix — This is the survey that was administered to all participants included in this study. (PDF) [file pone.0248858.s002.pdf]

Identification of Strategies that Increase PrEP Uptake for Adolescents and Young Adults in the Deep South

Parent Questionnaire

This **confidential** survey is being conducted by UAB Division of Adolescent Medicine. This survey is completely voluntary and anonymous and will be used to develop future educational tools regarding HIV prevention strategies and pre-exposure prophylaxis for HIV (PrEP).

Section 1:

1. AIDS is primarily a disease of white, gay men.
    a. True
    b. False
2. There is a cure for AIDS.
    a. True
    b. False
3. HIV is the virus that causes AIDS.
    a. True
    b. False
4. People who are HIV-positive don't always get AIDS.
    a. True
    b. False
5. AIDS is a medical condition in which your body cannot fight off diseases.
    a. True
    b. False
6. A person who is HIV-positive has AIDS.
    a. True
    b. False
7. HIV/AIDS impacts black Americans more than any other racial/ethnic group.
    a. True
    b. False
8. A mother who is HIV-positive can infect her child through breast milk.
    a. True
    b. False
9. Latex condoms are more effective than natural skin condoms in preventing HIV.
    a. True
    b. False
10. A person with HIV can spread it to others even before they get AIDS.
    a. True
    b. False
11. Having a sexually transmitted disease can increase a person's risk of getting HIV.
    a. True
    b. False
12. Using alcohol or drugs before or during sex can increase a person's risk of getting HIV.
    a. True
    b. False
13. "Unprotected or unsafe sex" means… (there may be more than one correct answer)
    a. Having sex with someone that you don't know.
    b. Having sex without using a condom/barrier.

    c. Having multiple sex partners.
    d. Having sex without using birth control (i.e., oral contraceptives).

14. Which of the following are ways that a person can get HIV? (there may be more than one correct answer)
    a. Hugging someone with AIDS.
    b. Sharing a needle to inject drugs with someone who is HIV-positive.
    c. Having anal sex without using a condom.
    d. Giving blood.
    e. Using a dirty toilet seat.
    f. Having oral sex without using a condom/barrier.
    g. Sharing an eating utensil with someone who is HIV-positive.
    h. Getting bitten by a mosquito that is carrying the virus.
    i. Having vaginal sex without using a condom.
15. Which of the following are ways that a person can reduce his/her risk of getting HIV?
    a. Withdrawing before ejaculation during sex without a condom/barrier.
    b. Not swallowing the discharge during oral sex without a condom/barrier.
    c. Using a condom/barrier during sex with a regular partner.
    d. Having anal sex instead of vaginal sex.
    e. Using a condom/barrier during sex with casual partners.
    f. Having sex without a condom/barrier, but reducing the number of partners.
    g. Having oral sex instead of vaginal sex.
    h. Having sex only with women.
16. There is a medication that is 95-99% effective in reducing a person’s chance of getting HIV should they be exposed to it.
    a. True
    b. False

Section 2:

PrEP is a medication that prevents people from getting HIV. It is a pill that is taken the same time each day. PrEP is very effective when taken daily, but like any medication there are short term side effects, like upset stomach, loss of appetite, or mild headache, and possible long-term side effects that are no worse than daily aspirin use. It has been approved for adults by the U.S.FDA (Food and Drug Administration).

17. Had you heard of PrEP before today?
    a. Yes
    b. No
    c. Not sure
18. If a teen were at high risk of becoming infected with HIV, do you think he/she should take this medication?
    a. Yes
    b. No
19. If a teen were prescribed PrEP, do you think he/she would be responsible in taking it as prescribed without the assistance of an adult?
    a. Yes
    b. No
20. If a teen asked for your support with taking PrEP, would you support them?
    a. Yes
    b. No
21. If you answered yes to Question 20, in what manner would you support them? Circle all that apply.
    a. Money to help pay for medications

    b. Encouragement
    c. Reminders to take medication
    d. Rides to appointments

Section 3:

1. Taking an active role in my child's health care is the most important factor in determining his/her health.
    a. Disagree strongly
    b. Disagree
    c. Agree
    d. Agree strongly
2. I am confident I can tell a doctor the concerns that I have about my child's health, even when he or she does not ask.
    a. Disagree strongly
    b. Disagree
    c. Agree
    d. Agree strongly
3. I am confident that I can follow through on medical treatments I need to do for my child at home.
    a. Disagree strongly
    b. Disagree
    c. Agree
    d. Agree strongly
4. I have been able to help my child maintain the recommended lifestyle changes for his/her health.
    a. Disagree strongly
    b. Disagree
    c. Agree
    d. Agree strongly
5. I am confident I can figure out solutions when new situations arise with my child's health.
    a. Disagree strongly
    b. Disagree
    c. Agree
    d. Agree strongly
6. I am confident that I can help my child maintain lifestyle changes, even during times of stress.
    a. Disagree strongly
    b. Disagree
    c. Agree
    d. Agree strongly

Section 4:

1. What is your age?
    A. 30 or younger
    B. 31 - 35 years old
    C. 36 - 45 years old
    D. 46 - 54 years old
    E. 55 years old or older

2. What is your sex?
    A. Female
    B. Male
    C. Trans female
    D. Trans male

E. Gender nonconforming
F. Other

3. Are you Hispanic or Latino?
A. Yes
B. No

4. What is your race? (Select one or more responses.)
A. American Indian or Alaska Native
B. Asian
C. Black or African American
D. Native Hawaiian or Other Pacific Islander
E. White

5. What is the age of the child with you for a clinic appointment today?
A. 14 years old
B. 15 years old
C. 16 years old
D. 17years old
E. 18 years old
F. 19 years old
G. 20 years old
H. 21 years old

6. What is the sex of the child with you for today's appointment?
A. Female
B. Male

7. I know that at least one of my children who is between the ages of 14 to 21 is having sex.
a. Definitely agree
b. Agree
c. Unsure
d. Disagree
e. Definitely disagree

8. What type of insurance does the child with you have?
A. Medicaid, Plan First, Patient First
B. All Kids
C. Blue Cross/Blue Shield
D. Other insurance. Please specify _______________
E. No insurance
